# Multisensory assessment for hearing phenotypes

**DOI:** 10.1016/j.heares.2025.109434

**Published:** 2025-09-23

**Authors:** Andrea J. DeFreese, René H. Gifford, Iliza M. Butera, Katelyn A. Berg, Mackenzie A. Lighterink, Mark T. Wallace

**Affiliations:** a Department of Hearing and Speech Sciences, Vanderbilt University, USA; b Hearts for Hearing, USA; c Vanderbilt Brain Institute, Vanderbilt University, USA; d Department of Psychology, Vanderbilt University, USA

**Keywords:** Hearing loss, Multisensory integration, Visual processing

## Abstract

Despite growing evidence of neural and behavioral plasticity following sensory loss, it remains unclear how multisensory processing varies across clinical hearing loss phenotypes. This study investigated visual perception and audiovisual (AV) integration in adults with varying degrees of hearing loss and hearing technology use. Participants included individuals with normal hearing (NH), hearing aid (HA) users, cochlear implant (CI) candidates, and CI users. To assess visual and multisensory processing, we administered a visual temporal order judgment (vTOJ) task, the McGurk illusion, a monosyllabic lipreading task, and an AV word recognition task. Results revealed a trend toward improved visual temporal resolution with increasing hearing loss severity, though this was confounded by age. McGurk illusion responses indicated that the presence of hearing loss decreased auditory weighting, while the severity of hearing loss increased visual weighting. Lipreading performance significantly improved as hearing loss progressed, with CI users outperforming all other groups—possibly due to the use of rehabilitation exercises in the CI clinical protocol. In contrast, AV benefit did not vary systematically with hearing loss, but showed a significant effect of age. Together, these findings suggest that visual performance and visual sensory weighting—more than AV integration per se—are modulated by hearing loss. These differences may reflect underlying plasticity of the cortical regions responsible for processing multisensory input. Furthermore, these findings highlight the potential utility of visual tasks in characterizing sensory phenotypes and informing clinical decision-making for individuals with hearing loss.

## 1. Introduction

Human perception relies on the synthesis of input from independent sensory modalities, a process known as multisensory integration (Stein and Stanford, 2008). In spoken communication, this integration allows listeners to combine auditory and visual speech cues based on their temporal and spatial alignment. The benefits of audiovisual (AV) integration are particularly evident in challenging listening environments, where visual cues can significantly enhance speech intelligibility (Sumby and Pollack, 1954). Audiovisual integration is particularly beneficial for individuals with hearing loss, who often rely more heavily on visual speech information to compensate for weakened auditory input. Indeed, there is evidence that individuals with hearing loss integrate audio and visual speech cues more effectively than those with normal hearing (Rouger et al., 2007).

In addition to behavioral changes, this enhanced integration may also stem from neural adaptations following sensory deprivation. Studies have shown that regions of the brain typically dedicated to the lost modality—such as the auditory cortex—can be recruited for processing other senses, such as visual motion (e.g., Doucet et al., 2006; Neville and Lawson, 1987). This sensory reorganization, known as crossmodal plasticity, has been documented in deaf individuals as auditory cortex activation in response to visual stimuli (Finney et al., 2001). This neural reorganization is also associated with enhanced visual processing abilities that can persist even after auditory input is restored via rehabilitation technologies (Bavelier et al., 2006; Callan et al., 2003; Merabet and Pascual-Leone, 2010; Shiell et al., 2014).

A compelling behavioral demonstration of the perceptual integration of auditory and visual speech cues is the McGurk effect, in which the presentation of incongruent auditory and visual syllables (e.g., an auditory /ba/ paired with a visual /ga/) can yield varied perceptual responses. While some individuals perceive the auditory speech token, others perceive the visual, and still others perceive an illusory fused percept (McGurk and MacDonald, 1976). Individuals with hearing loss often rely more on the visual component in this task whereas individuals with normal hearing tend to report the fused (audiovisual) percept (Huyse et al., 2013; Rouger et al., 2007; Tremblay et al., 2010). Despite growing evidence of behavioral and neural plasticity associated with sensory loss, our understanding of how these changes manifest across distinct clinical populations remains limited (Stevenson et al., 2017). Specifically, it is unclear whether various facets of multisensory integration can help distinguish among hearing loss phenotypes, such as those defined by degree of hearing loss and use of hearing technology.

The present study aims to characterize visual performance and audiovisual integration in adults with varying degrees of hearing loss and diverse hearing technology use. Participants included individuals with normal hearing (NH), hearing aid (HA) users, cochlear implant (CI) candidates, and CI users. To assess behavioral differences in visual processing among these groups, we employed two complementary visual perception paradigms. The first was a visual temporal order judgment (vTOJ) task, which measures low-level visual temporal processing independent of speech. The second was a lipreading task, which reflects speech-specific visual processing and is directly relevant to audiovisual communication. We also administered an audiovisual (AV) word recognition task to assess participants’ ability to integrate auditory and visual speech cues, and to quantify the perceptual benefit derived from multisensory integration. Finally, the McGurk illusion was used to evaluate the relative weighting of auditory and visual inputs in speech perception.

We hypothesized that visual performance, visual weighting, and AV integration increase with the severity of hearing loss (i.e., NH < HA < CI candidates/users), reflecting greater reliance on visual input. However, no significant differences are expected between CI candidates and CI users, as underlying neural adaptations may persist despite auditory restoration. Given the exploratory nature of this study, an alternative null hypothesis is also plausible, such that behavioral measures of visual processing and AV integration abilities do not vary systematically across degrees of hearing loss. This would suggest that the behavioral measures employed may lack sensitivity to detect underlying neural changes associated with auditory deprivation, or that multisensory integration is shaped more by individual-level factors rather than hearing status alone.

If significant behavioral differences do emerge across groups, these findings could lay the groundwork for using multisensory phenotyping to inform clinical decision-making. For example, if a hearing aid user demonstrates an AV integration profile more closely aligned with that of a CI candidate, it may indicate that neural changes are limiting the effectiveness of acoustic amplification. In such cases, earlier consideration of implantation could be warranted. More broadly, characterizing multisensory processing profiles may help inform intervention and hearing technology recommendations, supporting a more personalized approach to auditory rehabilitation.

## 2. Methods

### 2.1. Participants

To compare multisensory perception across a range of hearing statuses, this study included four groups of participants (Table 1). Across all groups, participants were native English speakers with reported normal or corrected-to-normal vision and no reported history of psychiatric or neurological disorders. This study was approved by the Institutional Review Board and informed consent was obtained from all participants (IRB# 101695). For each participant, duration of hearing loss was defined as the time between first diagnosis of hearing loss and date of testing.

#### 2.1.1. Normal hearing listeners

Participants included 28 normal hearing individuals (75 % female), who had pure tone thresholds </=25 dB HL at 250, 500, 1000, 2000, 3000, and 4000 Hz.

#### 2.1.2. Hearing aid users

Participants included 39 post-lingually deafened individuals (46 % female) with symmetric bilateral sensorineural hearing loss. These patients had at least one severe (>/=70 dB HL) unaided air conduction threshold and had been fit with bilateral hearing aids for at least six months. Importantly, while these HA users had at least one severe hearing threshold, indicating they were approaching CI candidacy, they did not yet meet labeled audiometric indications for adult CI candidacy (Zeitler et al., 2024). During testing, all participants wore their personal bilateral hearing aids that were found to be a good match to NAL-NL2 targets in the test box via visual inspection.

#### 2.1.3. Cochlear implant candidates

Participants included 22 peri- (*N* = 2) and post-lingually (*N* = 20) deafened individuals (42 % female) with bilateral hearing loss who met CI candidacy criteria in at least one ear, as determined by the clinical audiology department at our center. While patients were at least unilateral CI candidates, all patients had bilateral hearing loss that warranted bilateral hearing aid use. That is, no individuals with single-sided deafness were included in the CI candidate group. Therefore, during testing all participants wore bilateral hearing aids that were found to be a good match to NAL-NL2 targets in the test box via visual inspection.

#### 2.1.4. Cochlear implant users

Participants included 29 adult cochlear implant users who were peri-lingually (*N* = 3) or post-lingually (*N* = 26) deafened (69 % female; 25 with unilateral CIs, 3 with bilateral CIs). All CI users had at least six months of device use. During testing, all participants were assessed in their best-aided listening condition. For 9 individuals, this was a bimodal configuration with an electric-only CI and a contralateral hearing aid. For 11 individuals, this was an electric and acoustic stimulation (EAS) bimodal configuration, with CI in one ear and acoustic amplification (hearing aids) in both ears. For 6 individuals, this involved a unilateral CI with either normal hearing (*N* = 3) or unaided hearing loss (*N* = 3) in the contralateral ear. For 3 individuals, the best-aided condition included bilateral CIs, one of whom had bilateral EAS stimulation.

### 2.2. Procedures

In this study, all participants completed behavioral testing in a double-walled sound booth. During this testing, vTOJ, McGurk, and AV word recognition tests were completed. See Table 2 for descriptions of study tasks and outcome variables. All testing was completed in a single day following verification of hearing status (Table 2).

#### 2.2.1. Visual temporal order judgement

Visual temporal processing was examined using a temporal order judgment (TOJ) task, wherein two brief circles were presented in rapid succession at varying stimuli onset asynchronies (SOAs). Stimuli were generated using a Psychtoolbox extension (Brainard, 1997) in MATLAB (2006a). These white circles flashed (10-ms duration) either above or below a white fixation cross in the middle of a black screen. All stimuli were presented on a CRT monitor (100 Hz refresh rate) positioned 50 cm from participants. Individuals reported which stimuli occurred first by pressing either one (top) or two (bottom) on a standard keyboard. Visual behavioral performance was measured from this task by calculating the discrimination threshold for which each participant achieved 75 % accuracy, halfway between lowest (50 %; chance) and highest accuracy (100 %; Stevenson et al., 2013; Ryan A. Stevenson et al., 2014). Thresholds ≥150 ms, the largest SOA, were excluded from analysis as an indication that the participant could not complete the task.

#### 2.2.2. McGurk illusion

To assess perceptual sensory weighting, we used the multisensory McGurk illusion (Butera et al., 2023, 2022; Ryan A Stevenson et al., 2014). For this task, participants were presented with either congruent or incongruent auditory and visual speech stimuli. In the incongruent condition, participants may have perceived the auditory token, the visual token, or a novel fused percept that combines elements of both modalities (McGurk and MacDonald, 1976).

In this paradigm, visual stimuli were presented using MATLAB (2008a) and the Psychophysics Toolbox extensions. Visual stimuli were displayed on a CRT monitor positioned approximately 50 cm from the participant. There were 20 trials with incongruent auditory and visual information. Each trial began with a white fixation cross on a black background, followed by a 2 s video of a female articulating the syllables “ba” or “ga” (Stevenson et al., 2012). Auditory stimuli—also consisting of the syllables “ba” and “ga”—were presented at 65 dB SPL via a loudspeaker at zero degrees azimuth located one meter in front of the participant. Participants were presented with incongruent audiovisual speech tokens and were asked, “What did you hear?” Responses were made via a keypad with four options: “ba,” “ga,” “da,” or “tha.” The probability of perceiving the McGurk illusion was defined as the proportion of “da” and “tha” responses in the 20 trials, hereafter collectively referred to as “da” to denote the fused response.

#### 2.2.3. AV word recognition

To quantify AV integration in CI candidates, aided auditory, visual, and AV speech recognition in noise were assessed. According to the principle of inverse effectiveness (Holmes, 2007), degrading auditory performance increases AV gain. To maximize this gain, four-talker babble of female speakers was added to the target stimuli, which consists of monosyllabic words spoken by a female speaker. Since maximal AV gain occurs when auditory-only speech recognition performance is degraded to approximately 20 % accuracy (Ross et al., 2007), each participant’s auditory-only performance was first measured to help determine the specific signal-to-noise ratio (SNR) at which they would be expected to achieve maximal AV gain using a modified method of constant stimuli. First, three different SNRs, ranging from −10 to +15 dB, at which the researcher estimated the participant would correctly recognize between 0 % and 50 % of the words were selected. Clinical speech recognition scores in quiet, when available, where utilized to help select these three levels. Participants were then presented the target monosyllabic words at 60 dB SPL, in three different levels of four-talker babble (i.e., 20 words at each of the three selected SNRs). Performance was scored for word recognition accuracy at each of the three separate SNRs. The performance at these three SNRs was then used to approximate the participant’s psychometric function, which was, in turn, used to estimate the SNR at which the participant would have approximately 20 % word recognition accuracy.

After estimating the SNR for 20 % word recognition accuracy, auditory, visual, and AV speech comprehension was tested at that SNR. Testing consisted of 40-word lists of stimuli from the same target female speaker, also presented at 60 dB SPL. Participants were presented with either a still photo of the female speaker along with the word spoken in noise (auditory only), a video of the female speaker’s articulations without audio of the target word (visual only), or a video of the female speaker saying the word in noise including audio of the target word (AV). Word lists and recordings, matched for intelligibility in noise, were created by Picou and colleagues (Picou et al., 2011). All stimuli were presented through EPrime 2.0 using an external monitor and a centrally located loudspeaker, both positioned 60 cm from participant seated in a sound attenuated booth. Participants were instructed to repeat what they thought the target speaker said aloud and the experimenter confirmed the participant’s response by sharing the typed response on the monitor. All trials were scored for word recognition accuracy for each condition (auditory only, visual only, and AV). Since CI users and NH listeners participated as part of a larger study, they completed testing at multiple SNRs, but only the SNR with auditory only performance closest to 20 % was included in the current study.

AV benefit, intended to quantify multisensory integration, was calculated by comparing performance in the AV condition to the participant’s better-performing unisensory modality, while accounting for ceiling effects in the word recognition task. Specifically, AV benefit was calculated using the following equation: $AV\;benefit\;=\frac{\left(AV-max_{unisensory}\right)}{\left(100-max_{unisensory}\right)}⋆100$. This formula reflects the proportion of possible improvement gained from AV stimulation, relative to the remaining room for improvement beyond the best unisensory score.

### 2.3. Analytic plan

For vTOJ and AV word recognition analyses, separate analyses of covariance (ANCOVAs) were conducted with hearing group as a between-subjects factor and age included as a covariate to control for its potential influence. Where significant main effects of group were observed, post hoc pairwise comparisons of estimated marginal means were conducted using the Benjamini-Hochberg False Discovery Rate (FDR) procedure to correct for multiple comparisons. For analyses involving multiple conditions (e.g. multiple SOAs for vTOJ), FDR correction was also applied across conditions to control for inflation of type I error. All data were examined for normality using skewness and kurtosis measures prior to analysis.

Since McGurk auditory, visual and audiovisual responses violate the assumption of independence, a multinomial logistic regression was employed instead of an ANCOVA. This approach analyzed raw counts of the three response categories (AV, auditory, and visual) across participant groups, while controlling for age. The model was fit with response counts as the dependent variable and group and age as independent variables. The auditory response category served as the reference level. Predicted probabilities for each response category were extracted for each participant to obtain age-adjusted expected response patterns. To assess differences between groups for each response type, pairwise comparisons were conducted on the predicted probabilities using Wilcoxon rank-sum tests, with p-values adjusted for multiple comparisons via the FDR method. All analyses were conducted in R using pairwise deletion for missing data.

## 3. Results

### 3.1. Group demographics

A one-way ANOVA revealed a significant effect of group on age (F(3, 126) = 12.68, *p* < .001, η^2^ = .23), indicating that age significantly differed across the four groups. Post hoc pairwise comparisons using the FDR correction revealed that HA users were significantly older than CI users (*p* < .0001), CI candidates (*p* = .0004), and NH listeners (*p* < .0001; Table 1). No significant age differences were observed between CI users, CI candidates, and NH listeners (all *p’*s > .17). Given the significant differences in age across groups, all subsequent group-level analyses included age as a covariate to control for its potential confounding effects.

A Kruskal-Wallis test indicated a significant effect of group on degree of hearing loss, even when excluding NH listeners (χ^2^(2) = 12.62, *p* = .002). Follow-up pairwise Wilcoxon rank-sum tests with FDR correction revealed that HA users had significantly shorter durations of hearing loss than CI users (*p* = .029) and CI candidates (*p* = .005) The difference between CI users and CI candidates was not statistically significant (*p* = .074). While duration of hearing loss significantly differed across groups, it was not included in subsequent analyses as duration of hearing loss was fixed for the NH listeners (zero).

### 3.2. Visual temporal order judgement

**Fig. 1.**
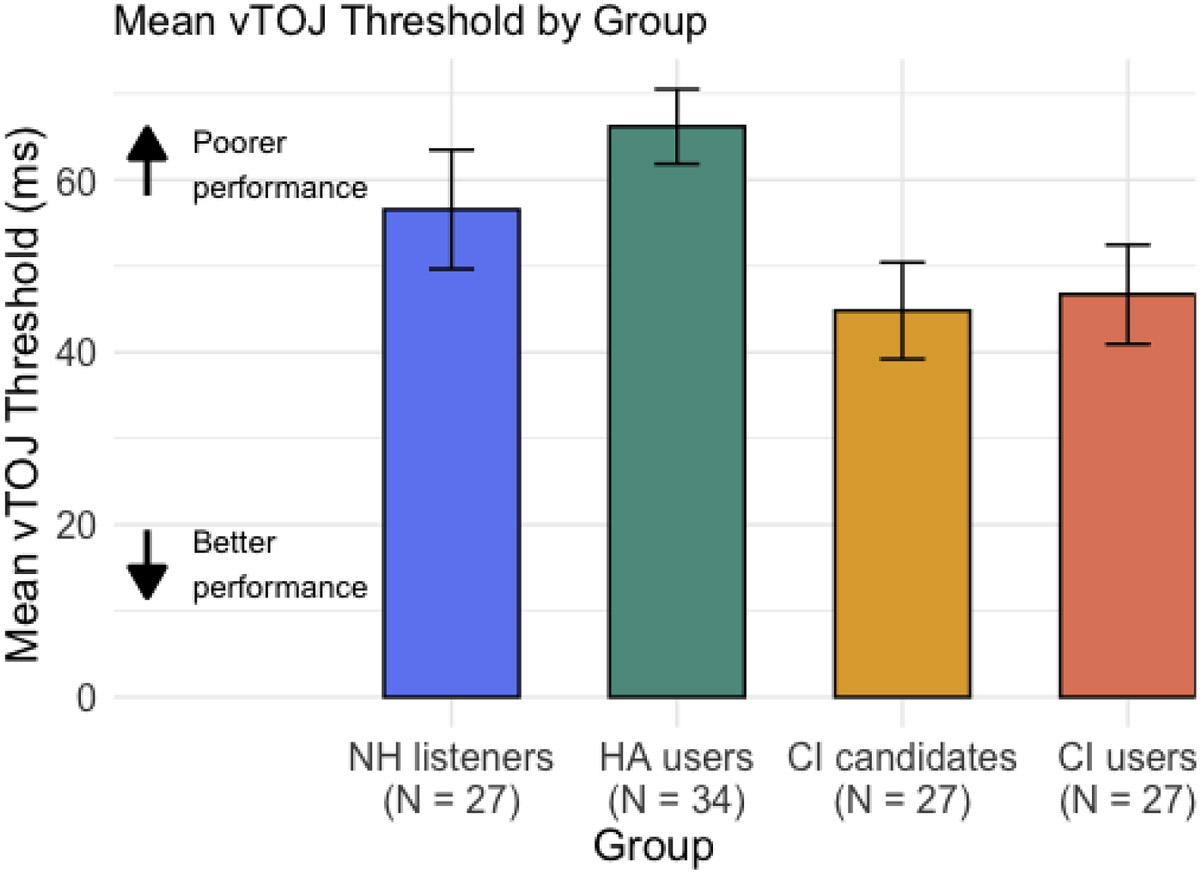
Visual Temporal Order Judgement Performance. Mean visual temporal order judgement threshold for 75 % correct in milliseconds by group with error bars for standard error. Higher vTOJ threshold corresponds to poorer performance, whereas lower vTOJ threshold corresonds to better performance. Note: ms = milliseconds; CI = cochlear implant; HA = hearing aid; NH = normal hearing.

Individuals who were unable to condition to the task (e.g., thresholds ≥150 ms) were present in each group (NH: 1; HA users: 5; CI candidates: 7; CI users: 1). These participants were excluded from all further vTOJ analyses, and group performance was subsequently compared. Across the four groups, HA users demonstrated the highest average vTOJ threshold (*M* = 66.2, SD = 25.3, *N* = 34), followed by NH listeners (*M* = 56.6, SD = 35.9, *N* = 27), CI users (*M* = 46.7, SD = 29.9, *N* = 27), and CI candidates (*M* = 44.8, SD = 29.1, *N* = 27; Fig. 1A). A one-way ANCOVA was conducted to examine differences in vTOJ thresholds across the four listener groups (NH listeners, HA users, CI candidates, and CI users), controlling for age. There was a significant main effect of group on vTOJ thresholds (F(3, 110) = 4.05, *p* = .009, partial η^2^ = .10), as well as a significant effect of age (F(1, 110) = 25.83, *p* < .001, partial η^2^ = .19). Post hoc pairwise comparisons of the age-adjusted group means were conducted using the FDR correction. None of the group comparisons reached statistical significance after correction (all *p*’s > .05), suggesting that the overall group effect reflects a distributed trend rather than strong differences between specific pairs. Visual inspection of the age-adjusted means suggested numerically higher thresholds for HA users compared to the other groups.

### 3.3. McGurk illusion

A multinomial logistic regression was conducted to examine whether response type (i.e., auditory, AV, visual) in the incongruent McGurk task significantly differed across groups (CI users, CI candidates, HA users, and NH listeners), while controlling for age. The auditory response served as the reference category. The overall model indicated a significant effect of both age and group on the likelihood of reporting AV and visual responses relative to auditory responses. Age was significantly associated with an increased likelihood of reporting both AV and visual percepts compared to auditory percepts. For each additional year of age, the odds of reporting an AV percept (vs auditory-only) increased by approximately 7.5 % (*β* = 0.073, *p* < .001), and the odds of reporting a visual-only percept increased by 4.9 % (*β* = 0.048, *p* < .001). Regarding the effect of group, compared to CI users, NH listeners were significantly less likely to report AV (*β* = −2.27, *p* < .001) and visual (*β* = −3.81, *p* < .001) percepts. HA users also showed reduced odds of AV (*β* = −1.34, *p* = .003) and visual (*β* = −1.48, *p* < .001) responses. CI candidates did not differ significantly from CI users (*p* > 0.05). Together, these findings indicate that the interaction between group and response type was statistically significant, confirming that response profiles differed across groups.

**Fig. 2.**
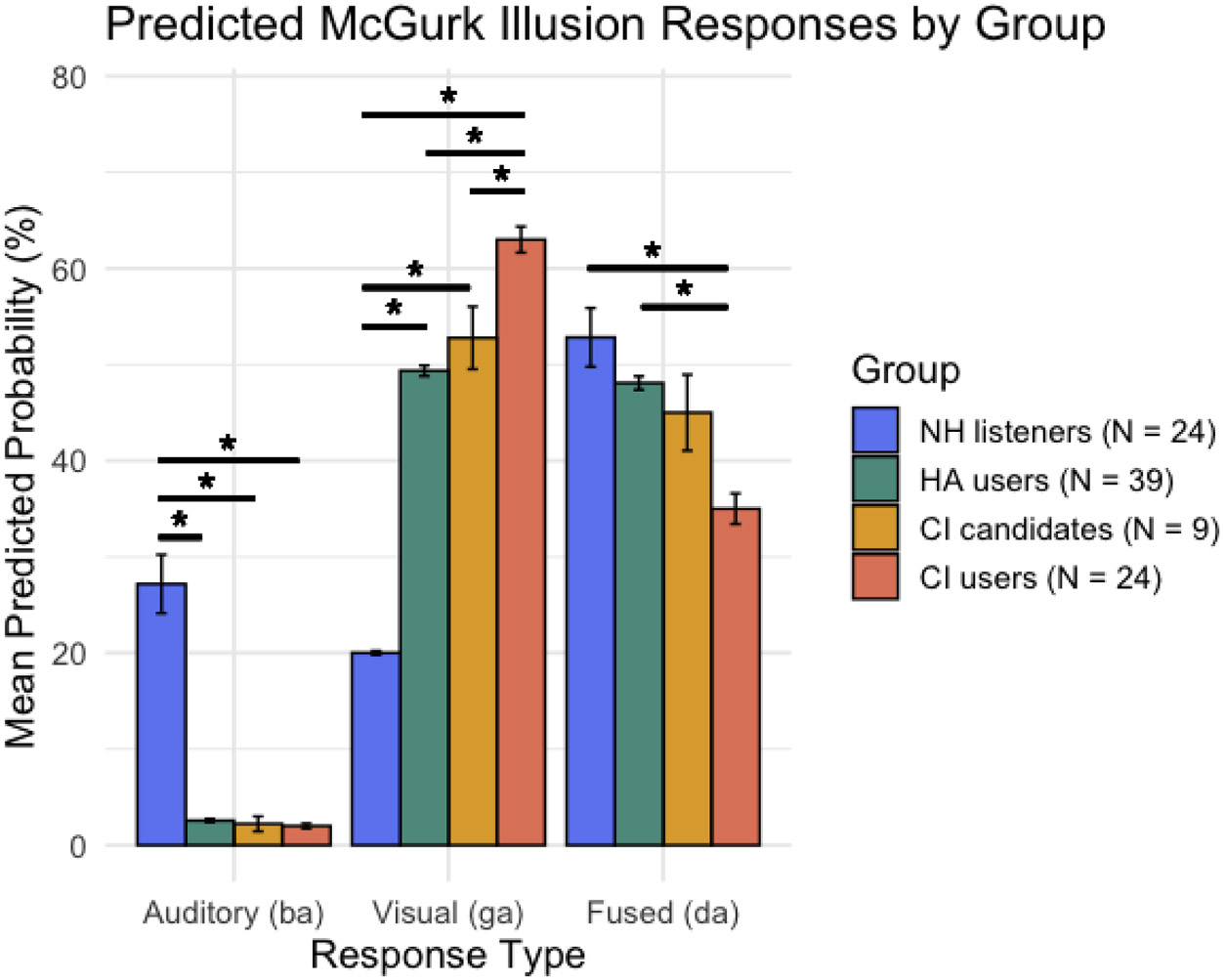
McGurk Illusion Responses. Predicted percentage probability of perceived responses for the auditory, visual and fused (audiovisual) syllables by group. *Note:* CI = cochlear implant; HA = hearing aid; NH = normal hearing; * = *p* < 0.05.

Group-wise predicted probabilities revealed notable differences in response profiles. Specifically, CI users showed a mean predicted probability of 2.0 % for auditory responses, 35.0 % for AV responses, and 63.0 % for visual responses. CI candidates exhibited similar patterns, with 2.2 % auditory, 45.0 % AV, and 52.8 % visual responses. HA users showed 2.6 % auditory, 48.1 % AV, and 49.4 % visual responses. In contrast, NH listeners displayed a markedly different profile, with 27.2 % auditory, 52.8 % AV, and 20.0 % visual responses (Fig. 2).

Post-hoc pairwise Wilcoxon comparisons revealed that NH listeners reported the auditory percept significantly more than all other groups (*p’*s < .001), while CI users, CI candidates, and HA users did not differ from one another in this regard, with all groups showing predicted probabilities below 3 %. For visual responses, CI users showed significantly greater visual percept rates compared to CI candidates (*p* = .008), HA users (*p* < .001), and NH listeners (*p* < .001). CI candidates also reported more visual responses than NH listeners (*p* < .001) but did not differ significantly from HA users (*p* = .195). For AV responses, CI users reported significantly fewer percepts than HA users (*p* < .001) and NH listeners (*p* < .001), though the difference between CI users and CI candidates was not significant (*p* = .052). No other pairwise comparisons for AV responses reached significance (*p*’s > 0.05).

### 3.4. AV word recognition

Separate one-way ANCOVAs were conducted to examine differences in auditory-only, visual only, and audiovisual word recognition across four listener groups (NH listeners, HA users, CI candidates and CI users), controlling for age. Separate analyses were utilized because auditory-only performance was deliberately fixed at approximately 20 % (Ross et al., 2007) to achieve maximal AV gain, which constrained the variance in this condition across all groups. Conversely, visual-only and audiovisual conditions, which were not fixed and were the primary focus for examining group differences in visual speech processing abilities, were more variable. To correct for the multiple pairwise comparisons made in each condition, the FDR method was utilized. Since auditory-only performance was fixed at approximately 20 %, each participant was tested at an individualized SNR. SNRs ranged from −10 dB to 15 dB, with an average of −7 dB for NH listeners, −1 dB for HA users, 7 dB for CI candidates, and 3 dB for CI users. A one-way ANOVA revealed a significant effect of group on SNR (F(3, 121) = 50.31, *p* < .001), indicating that SNR significantly differed across the four groups. For auditory-only word recognition performance at these SNRs, there was no significant difference across groups (F(3, 120) = 1.21, *p* = .308, partial η^2^ = .03) or age (*F*(1120) = 0.005, *p* = .941, partial η^2^ = .00005), with mean scores around the projected 20 % (NH: *M* = 27.0 %, SD = 10.8 %, *N* = 26; HA: *M* = 24.6 %, SD = 12.7 %, *N* = 39; CI: *M* = 22.3 %, SD = 9.96 %, *N* = 26; and CI candidates: *M* = 21.5 %, SD = 13.3 %, *N* = 34). This between group similarity suggests that auditory-only performance across groups was successfully controlled for by the study design.

**Fig. 3.**
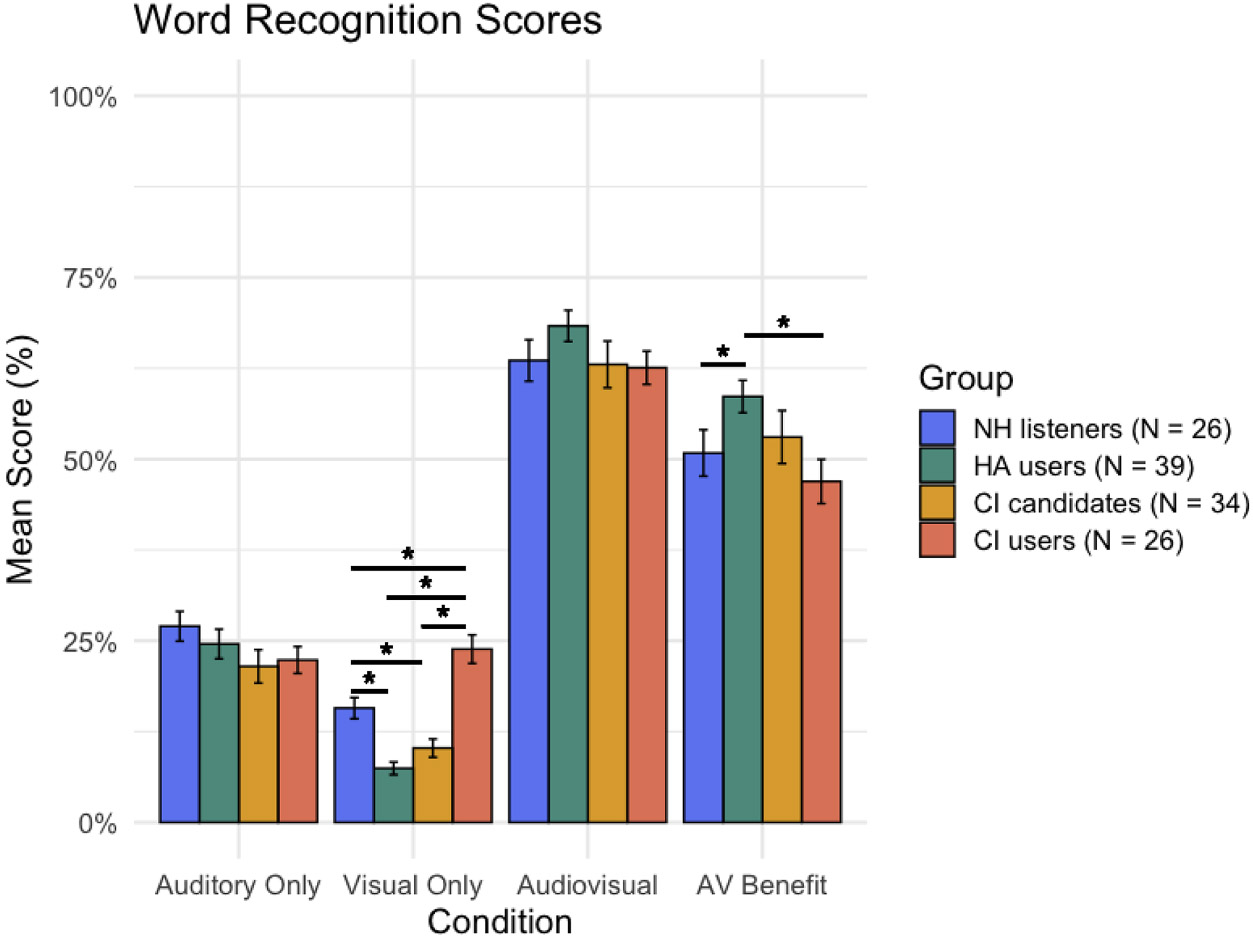
Word Recognition Performance. Mean word recognition score ( %) for the auditory only, visual only, and audiovisual speech tasks with error bars representing the standard error. These performances were then used to calcualte the audiovisual benefit. *Note:* AV = audiovisual; CI = cochlear implant; HA = hearing aid; NH = normal hearing; * = *p* < 0.05.

Visual-only word recognition (i.e. lipreading) significantly differed by group (F(3, 120) = 30.20, *p* < .001, partial η^2^ = .43) and age (F(1, 120) = 18.12, *p* < .001, partial η^2^ = .13). On average, performance was highest in the CI Users (*M* = 23.8 %, SD = 10.5 %, *N* = 26), followed by the NH listeners (*M* = 15.7 %, SD = 7.70 %, *N* = 26), CI candidates (*M* = 10.2 %, SD = 7.33 %, *N* = 34), and HA users (*M* = 7.44 %, SD = 5.49 %, *N* = 39). Post hoc pairwise comparisons using estimated marginal means with FDR correction revealed that CI users performed significantly better than CI candidates (*p* < .001), HA users (*p* < .001), and NH listeners (*p* < .001). NH listeners also performed significantly better than CI candidates (*p* = .0394) and HA users (*p* = .0394; Fig. 3). No other pairwise group differences reached statistical significance (*p*’s > .05).

Audiovisual word recognition did not significantly differ by group (F (3, 120) = 1.15, *p* = .333, partial η^2^ =.03). HA (*M* = 68.3 %, SD = 13.4 %, *N* = 39), NH (*M* = 63.6 %, SD = 15.1 %, *N* = 26), CI candidates (*M* = 63.0 %, SD = 18.7 %, *N* = 34), and CI (*M* = 62.6 %, SD = 12.3 %, *N* = 26; Fig. 3). Audiovisual word recognition did, however, significantly differ by age (F(1, 120) = 6.35, *p* = .013, partial η^2^ = .05).

To quantify audiovisual integration, AV benefit was calculated by comparing the strongest unisensory modality performance (auditory only or visual only) to the AV word recognition performance. For most participants the strongest unisensory modality was the auditory condition (*n* = 98); however, for a portion of participants, the strongest unisensory modality was found to be the visual condition (*n* = 27). An ANCOVA revealed a marginally significant effect of group on AV benefit (F(3, 120) = 2.67, *p* = .051, partial η^2^ = .06), and a significant effect of age (F(1, 120) = 5.51, *p* = .021, partial η^2^ = .04). On average, AV benefit was highest in the HA group (*M* = 58.6 %, SD = 13.9 %, *N* = 39), followed by CI candidates (*M* = 53.0 %, SD = 21.3 %, *N* = 34), NH listeners (*M* = 50.8 %, SD =16.9 %, *N* = 26), and CI users (*M* = 46.9 %, SD = 16.4 %, *N* = 26). Post hoc pairwise comparisons using estimated marginal means with FDR correction revealed the HA users had significantly greater AV benefit than CI users (*p* = .0041) and NH listeners (*p* = .0262; Fig. 3). No other group comparisons were statistically significant (*p*’s > .05).

## 4. Discussion

The primary aim of this study was to characterize visual abilities and multisensory integration in individuals with varying degrees of hearing loss and differing use of hearing technology. To account for differences in both hearing thresholds and functional communication ability, we examined four distinct groups: NH listeners, HA users, CI candidates, and CI users. Visual abilities were assessed through a visual temporal order task and a lipreading task, while AV processing was measured using the McGurk illusion and an AV word recognition task. Group differences were evaluated using ANCOVAs and a multinomial logistic regression, with age as a covariate, as appropriate. The findings suggest that visual performance and visual weighting—more than AV processing—are influenced by hearing loss, offering preliminary support for the use of visual tasks in characterizing sensory phenotypes and informing intervention strategies.

### 4.1. Trend toward better visual temporal processing with greater hearing loss

Despite the lack of significant group differences in visual temporal processing (measured via the vTOJ task), age-adjusted means suggest that CI users and candidates may exhibit enhanced temporal resolution compared to NH listeners (Fig. 1A). This pattern is consistent with prior research reporting improved visual temporal processing in adults with hearing loss (Butera et al., 2018) and provides tentative support for the hypothesis that visual performance improves with increasing hearing loss severity. Interestingly, however, HA users in the present study demonstrated the poorest (i.e., highest) visual temporal processing thresholds. This contradicts the aforementioned relationship between vTOJ threshold and hearing loss. However, this contradictory pattern seen in the HA users is likely confounded by age, as HA users were significantly older than the other hearing groups, and age was independently associated with poorer vTOJ. Despite this potential confound, there remains an observable difference in vTOJ thresholds between HA users and CI candidates. While not significant, this trend may reflect functional communication differences between individuals who benefit from a HA and those who qualify for a CI. Replication with better age-matched groups is needed to clarify the significance of this relationship, if any. If this trend proves to be significant in future studies, it would support the potential utility of the vTOJ task as a quick, language-independent tool for evaluating CI candidacy—particularly valuable for patients who cannot complete standard speech recognition tests.

To further examine this group level trend in vTOJ thresholds, post hoc signal detection theory (SDT) analyses were completed using previously published techniques (Butera et al., 2018). These analyses found no significant group differences in either lower-level sensory processing (sensitivity) or higher-level sensory processing (response bias). These findings suggest that the observed group-level differences in vTOJ thresholds are unlikely to be driven solely by either bottom-up or top-down processes, as indexed by sensitivity and response bias, respectively. Instead, the pattern may reflect contributions from both levels of processing, or other mechanisms not fully captured by SDT. It should also be noted that several individuals could not condition to the task (e.g., thresholds ≥150 ms). These individuals were present in each group (NH: 1; HA users: 5; CI candidates: 7; CI users: 1), indicating that there was no group level effect. Further investigation into lack of conditioning is warranted before replication of this task in a larger clinical population.

### 4.2. Presence—not severity—of hearing loss alters sensory weighting

The McGurk illusion, a sensory task that introduces incongruent auditory and visual stimuli, was employed to probe sensory weighting. Both age and group were found to have a significant effect on visual and AV responses, relative to the reference auditory response, suggesting these factors influence sensory weighting. Specifically, as age increased, so did the likelihood of reporting both visual and AV percepts compared to auditory percepts. Although modest, this effect aligns with prior studies across broader age ranges (Pepper and Nuttall, 2023; Sekiyama et al., 2014; Setti et al., 2013), underscoring that age influences audiovisual binding even within a relatively age-controlled cohort.

Regarding the effect of group on unisensory reports, NH listeners reported significantly more auditory percepts than all other groups, with a predicted probability of 27 %. Conversely, CI users, CI candidates, and HA users did not differ significantly among themselves, all showing auditory response probabilities below 3 %. This implies that the presence of hearing loss, rather than the degree or hearing technology utilized, influences auditory percept rates. Conversely, visual percept rates increased progressively with hearing loss severity (as proxied by hearing technology: NH < HA < CI candidates < CI users), with the exception of no significant difference between HA users and CI candidates. The absence of difference between these two groups suggests sensory weighting may not directly reflect functional communication changes accompanying progression to CI candidacy. Alternatively, this could in part be driven by differences in hearing technology used, as both the HA users and CI candidates wore traditional hearing aids; whereas the other two groups had normal hearing or a CI. Overall, the pattern of increasing reliance on visual stimuli with greater hearing loss supports the hypothesis that weighting of visual stimuli increases with hearing loss severity (Butera et al., 2023; Stropahl and Debener, 2017). Future research should examine individuals with milder hearing loss to identify the threshold at which sensory reweighting emerges and the precise influence of hearing loss severity.

Regarding fused AV syllable (McGurk illusion) perception, CI users reported significantly fewer AV percepts than HA users and NH listeners, while the difference between CI users and CI candidates approached significance. Interestingly, however, we found no significant difference between HA users and CI candidates’ perception of the AV stimulus. This finding diverges from prior evidence showing that McGurk susceptibility is negatively correlated with hearing aid benefit—a distinction that should theoretically separate CI candidates from HA users—and is associated with decreased functional connectivity between auditory cortex and fusiform gyrus (Rosemann et al., 2021). The absence of this behavioral distinction in the present study suggests that the McGurk task may be less sensitive than neural measures for detecting subtle group differences. Alternatively, the null result could reflect smaller sample sizes and age differences between HA users and CI candidates in the present study (Fig. 2). Despite these caveats, the broader group trends suggest that sensory weighting shifts as individuals progress toward CI candidacy. This raises the possibility that sensory weighting, as measured by the McGurk task, could serve as a quick screening tool for CI referral decisions. Similar to trends observed in vTOJ thresholds, however, further replication is necessary to establish its clinical utility.

### 4.3. Lipreading performance largely mirrors hearing loss severity

Visual-only word recognition (lipreading) also significantly differed across groups, with CI users performing significantly better than CI candidates, HA users, and NH listeners. This pattern supports the hypothesis that visual speech perception improves as auditory input deteriorates (Auer and Bernstein, 2007; Bernstein et al., 2022; Rouger et al., 2007). Contrary to this hypothesis, however, CI users also outperformed CI candidates—despite having comparable hearing thresholds and ages. This difference could reflect the effects of post-implantation aural rehabilitation, which often includes AV training components (Sato et al., 2020). This explicit training may not only be impacting auditory-only speech recognition, but also overall visual-only speech recognition. Another possibility is that CI users engage visual working memory resources to a greater extent than CI candidates, consistent with evidence that auditory working memory demands increase with degraded input that a CI provides (Rönnberg et al., 2013) and that CI users show compensatory reliance on visual working memory during speech perception (Moberly et al., 2017). Further investigation using a within group design is warranted to better elucidate the driver of this pre vs post implantation difference in visual only speech recognition performance.

Contrary to the trend of poorer hearing being associated with better visual-only speech recognition, HA users performed significantly worse than NH listeners. This unexpected finding may be partially explained by age differences, as HA users were significantly older than participants in all other hearing groups. Differences in test administration may also have contributed. Specifically, HA users and CI candidates were tested with the same single phonemically balanced word list, while NH listeners and CI candidates in a larger companion study were exposed to multiple word lists. As a result, both participant age and variability in stimulus exposure may have confounded the observed group differences in visual-only speech recognition performance.

### 4.4. AV integration may be impacted by hearing technology

AV benefit—measured by comparing the strongest unisensory score to multisensory performance—showed a marginal effect of hearing group and a significant effect of age. Interestingly, this effect of group was driven by the HA users, who experienced significantly greater AV benefit than CI users and NH listeners, but not CI candidates fit with the same hearing technology (Fig. 3). This finding challenges the assumption that CI users, with their enhanced lipreading performance, would also show superior AV integration. One possible explanation is that CI users may rely more on visual cues, NH listeners may rely on auditory cues, and HA users benefit from integrating both modalities. Neuroimaging evidence supports this interpretation, showing that visual speech activation in the left auditory cortex is associated with enhanced lipreading abilities in CI users (Anderson et al., 2019). If auditory cortex is dominated by visual inputs, adding a degraded CI signal may yield little additional benefit, whereas HA users still receive usable acoustic input, producing greater AV gain. Thus, reduced AV benefit in CI users may reflect a different weighting of sensory inputs shaped by cortical reorganization or differences in hearing technology (e.g. signal quality), rather than impaired integration. This is further supported by the McGurk illusion data, wherein CI users reported significantly fewer AV percepts (integration) than HA users (Fig. 2). To clarify the drivers of this marginal between-group difference, replication using within-group designs combined with neuroimaging is needed to determine whether CI use impairs—or merely alters—AV integration.

### 4.5. Limitations and future directions

Several limitations should be noted. First, as previously mentioned, HA users in this study were significantly older than other participants. While age was statistically controlled, this demographic skew remains a potential confound. Second, the HA group was restricted to individuals with at least one severe hearing threshold (≥70 dB HL), limiting generalizability to all HA users. This was a deliberate design choice to examine functional differences among groups with similar audiometric profiles, but differing interventions (HA, CI candidate, and CI user). Further replication with better age-matched participants and individuals with milder forms of hearing loss is warranted to extend these findings on HA users to a broader population.

While visual and audiovisual behavioral measures are often interpreted as proxies for underlying differences in sensory processing (Bavelier et al., 2006; Callan et al., 2003; Merabet and Pascual-Leone, 2010; Shiell et al., 2014), such conclusions remain speculative without direct quantification of neural response patterns. Consequently, the behavioral similarities observed in the present study may be masking underling neural differences among these groups. Therefore, future studies incorporating neuroimaging may offer the ability to observe the neural changes that occur following hearing loss and rehabilitation. These neural measures could enhance the precision of multisensory phenotyping and provide stronger evidence to guide clinical decision-making.

## 5. Conclusion

This study provides preliminary evidence that visual behavioral measures—particularly tasks assessing temporal processing, sensory weighting, and lipreading—may be more sensitive to changes associated with changes in hearing than traditional indices of auditory performance. While group-level differences were modest and often confounded by age, the observed patterns suggest that visual performance varies with hearing status and hearing technology—which may reflect functional differences relevant to intervention outcomes. Importantly, findings point to the potential clinical utility of visual and AV behavioral tasks (e.g. vTOJ or McGurk) for sensory phenotyping, particularly in cases where standard auditory assessments are not feasible. However, the lack of consistent statistical significance across groups highlights the need for replication in larger, better age-matched cohorts and inclusion of individuals with milder hearing loss. Future work incorporating neuroimaging may be more sensitivity to sensory processing differences amongst these groups, offering a more refined tools for tailoring auditory rehabilitation strategies.

## Figures and Tables

**Table 1:** Demographic information for participants by group.

Hearing Group	Age	Biological Sex	Duration of Hearing Loss
Normal Hearing (*N* = 28)	52.7 (33–71) years	7 male 21 female	–
Hearing Aid User (N = 39)	68.8 (55–79) years*	21 male 18 female	18.0 (1.17–73) years*
CI Candidate (*N* = 34)	57.2 (19–82) years	20 male 14 female	33.5 (0.5–74) years
CI User (*N* = 29)	52.7 (33–71) years	9 male 20 female	23.9 (2–56) years

* *p* < 0.05 for Tukey’s HSD (see Results). CI = cochlear implant

**Table 2:** In this study design, participants completed approximately 35 min of behavioral testing which included auditory, visual, and audiovisual tasks. SNR = signal to noise ratio; A = auditory; V = visual; AV = audiovisual; ms = milliseconds; vTOJ = visual temporal order judgement; SOA = stimulus onset asynchrony.

Task	Trials (total)	Schematic of Stimuli	Prompt	Output Measured
AV word recognition	40 words x 3 conditions x 1 SNRs (240)	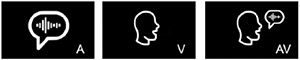	You will see and/or hear a woman saying words. Your job is to tell me what word she said.	% word accuracy; lipreading; % AV gain (ii)
vTOJ	10 runs x 16 SOAs (160)	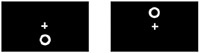	If the top flash came first, press 1. If the bottom flash came first, press 2.	threshold [ms]
McGurk	20 incongruent	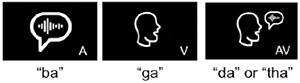	You will see and hear a woman saying syllables. Your job is to push the button as to whether you thought she said: “ba”, “ga”, “da”, or “tha.”	% auditory responses; % AV responses; % visual responses

## Data Availability

Data will be made available on request

## References

[R1] Anderson CA, Wiggins IM, Kitterick PT, Hartley DEH, 2019. Pre-operative brain imaging using functional near-infrared spectroscopy helps predict cochlear implant outcome in deaf adults. J. Assoc. Res. Otolaryngol 20, 511–528. 10.1007/s10162-019-00729-z. 31286300 PMC6797684

[R2] Auer ET, Bernstein LE, 2007. Enhanced visual speech perception in individuals with early-onset hearing impairment. J. Speech Lang. Hear. Res. JSLHR 50, 1157–1165. 10.1044/1092-4388(2007/080. 17905902

[R3] Bavelier D, Dye MWG, Hauser PC, 2006. Do deaf individuals see better? Trends Cogn. Sci 10, 512–518. 10.1016/j.tics.2006.09.006. 17015029 PMC2885708

[R4] Bernstein LE, Jordan N, Auer ET, Eberhardt SP, 2022. Lipreading: a review of its continuing importance for speech recognition with an acquired hearing loss and possibilities for effective training. Am. J. Audiol 31, 453–469. 10.1044/2021_AJA-21-00112. 35316072 PMC9524756

[R5] Brainard DH, 1997. The psychophysics toolbox. Spat. Vis 10, 433–436. 9176952

[R6] Butera IM, Stevenson RA, Mangus BD, Woynaroski TG, Gifford RH, Wallace MT, 2018. Audiovisual temporal processing in postlingually deafened adults with cochlear implants. Sci. Rep 8, 11345. 10.1038/s41598-018-29598-x. 30054512 PMC6063927

[R7] Butera IM, Larson ED, DeFreese AJ, Lee AK, Gifford RH, Wallace MT, 2022. Functional localization of audiovisual speech using near infrared spectroscopy. Brain Topogr. 35, 416–430. 10.1007/s10548-022-00904-1. 35821542 PMC9334437

[R8] Butera IM, Stevenson RA, Gifford RH, Wallace MT, 2023. Visually biased perception in cochlear implant users: a study of the mcgurk and sound-induced flash illusions. Trends Hear. 27, 23312165221076681. 10.1177/23312165221076681. 37377212 PMC10334005

[R9] Callan DE, Jones JA, Munhall K, Callan AM, Kroos C, Vatikiotis-Bateson E, 2003. Neural processes underlying perceptual enhancement by visual speech gestures. Neuroreport 14, 2213–2218. 10.1097/00001756-200312020-00016. 14625450

[R10] Doucet ME, Bergeron F, Lassonde M, Ferron P, Lepore F, 2006. Cross-modal reorganization and speech perception in cochlear implant users. Brain J. Neurol 129, 3376–3383. 10.1093/brain/awl264. 17003067

[R11] Finney EM, Fine I, Dobkins KR, 2001. Visual stimuli activate auditory cortex in the deaf. Nat. Neurosci 4, 1171–1173. 10.1038/nn763. 11704763

[R12] Holmes NP, 2007. The law of inverse effectiveness in neurons and behaviour: Multisensory integration versus normal variability. Neuropsychologia 45, 3340–3345. 10.1016/j.neuropsychologia.2007.05.025. 17663007

[R13] Huyse A, Berthommier F, Leybaert J, 2013. Degradation of labial information modifies audiovisual speech perception in cochlear-implanted children. Ear Hear. 34, 110–121. 10.1097/AUD.0b013e3182670993. 23059850

[R14] The MathWorks Inc. MATLAB version: 7.2.0 (R2006a). Natick, Massachusetts, United States: The MathWorks Inc.; 2006.

[R15] The MathWorks Inc. MATLAB version: 7.6.0 (R2008a). Natick, Massachusetts, United States: The MathWorks Inc.; 2008.

[R16] McGurk H, MacDonald J, 1976. Hearing lips and seeing voices. Nature 264, 746–748. 10.1038/264746a0. 1012311

[R17] Merabet LB, Pascual-Leone A, 2010. Neural reorganization following sensory loss: the opportunity of change. Nat. Rev. Neurosci 11, 44–52. 10.1038/nrn2758. 19935836 PMC3898172

[R18] Moberly AC, Houston DM, Harris MS, Adunka OF, Castellanos I, 2017. Verbal working memory and inhibition-concentration in adults with cochlear implants. Laryngoscope Investig. Otolaryngol 2, 254–261. 10.1002/lio2.90. PMC5655567 29094068

[R19] Neville HJ, Lawson D, 1987. Attention to central and peripheral visual space in a movement detection task: an event-related potential and behavioral study. II. Congenitally deaf adults. Brain Res. 405, 268–283. 10.1016/0006-8993(87)90296-4. 3567605

[R20] Pepper JL, Nuttall HE, 2023. Age-related changes to multisensory integration and audiovisual speech perception. Brain Sci. 13, 1126. 10.3390/brainsci13081126. 37626483 PMC10452685

[R21] Picou EM, Ricketts TA, Hornsby BWY, 2011. Visual cues and listening effort: individual variability. J. Speech Lang. Hear. Res. JSLHR 54, 1416–1430. 10.1044/1092-4388(2011/10-0154). 21498576

[R22] Rönnberg J, Lunner T, Zekveld A, Sörqvist P, Danielsson H, Lyxell B, Dahlström O, Signoret C, Stenfelt S, Pichora-Fuller MK, Rudner M, 2013. The ease of language understanding (ELU) model: theoretical, empirical, and clinical advances. Front. Syst. Neurosci 7, 31. 10.3389/fnsys.2013.00031. 23874273 PMC3710434

[R23] Rosemann S, Gieseler A, Tahden M, Colonius H, Thiel CM, 2021. Treatment of age-related hearing loss alters audiovisual integration and resting-state functional connectivity: a randomized controlled pilot trial. eNeuro 8. 10.1523/ENEURO.0258-21.2021.ENEURO.0258-21.2021. PMC8658542 34759049

[R24] Ross LA, Saint-Amour D, Leavitt VM, Javitt DC, Foxe JJ, 2007. Do you see what I am saying? Exploring visual enhancement of speech comprehension in noisy environments. Cereb. Cortex 17, 1147–1153. 10.1093/cercor/bhl024.N.Y.N1991. 16785256

[R25] Rouger J, Lagleyre S, Fraysse B, Deneve S, Deguine O, Barone P, 2007. Evidence that cochlear-implanted deaf patients are better multisensory integrators. Proc. Natl. Acad. Sci. U. S. A 104, 7295–7300. 10.1073/pnas.0609419104. 17404220 PMC1855404

[R26] Sato T, Yabushita T, Sakamoto S, Katori Y, Kawase T, 2020. In-home auditory training using audiovisual stimuli on a tablet computer: feasibility and preliminary results. Auris Nasus Larynx 47, 348–352. 10.1016/j.anl.2019.09.006. 31708168

[R27] Sekiyama K, Soshi T, Sakamoto S, 2014. Enhanced audiovisual integration with aging in speech perception: a heightened McGurk effect in older adults. Front. Psychol 5, 323. 10.3389/fpsyg.2014.00323. 24782815 PMC3995044

[R28] Setti A, Burke KE, Kenny R, Newell FN, 2013. Susceptibility to a multisensory speech illusion in older persons is driven by perceptual processes. Front. Psychol 4, 575. 10.3389/fpsyg.2013.00575. 24027544 PMC3760087

[R29] Shiell MM, Champoux F, Zatorre RJ, 2014. Enhancement of visual motion detection thresholds in early deaf people. PloS One 9, e90498. 10.1371/journal.pone.0090498. 24587381 PMC3938732

[R30] Stein BE, Stanford TR, 2008. Multisensory integration: current issues from the perspective of the single neuron. Nat. Rev. Neurosci 9, 255–266. 10.1038/nrn2331. 18354398

[R31] Stevenson RA, Zemtsov RK, Wallace MT, 2012. Individual differences in the multisensory temporal binding window predict susceptibility to audiovisual illusions. J. Exp. Psychol. Hum. Percept. Perform 38, 1517–1529. 10.1037/a0027339. 22390292 PMC3795069

[R32] Stevenson RA, Wilson MM, Powers AR, Wallace MT, 2013. The effects of visual training on multisensory temporal processing. Exp. Brain Res 225, 479–489. 10.1007/s00221-012-3387-y. 23307155 PMC3606590

[R33] Stevenson Ryan A, Ghose D, Fister JK, Sarko DK, Altieri NA, Nidiffer AR, Kurela LR, Siemann JK, James TW, Wallace MT, 2014. Identifying and quantifying multisensory integration: a tutorial review. Brain Topogr. 27, 707–730. 10.1007/s10548-014-0365-7. 24722880

[R34] Stevenson Ryan A., Siemann JK, Schneider BC, Eberly HE, Woynaroski TG, Camarata SM, Wallace MT, 2014. Multisensory temporal integration in autism spectrum disorders. J. Neurosci. Off. J. Soc. Neurosci 34, 691–697. 10.1523/JNEUROSCI.3615-13.2014. PMC3891950 24431427

[R35] Stevenson RA, Sheffield SW, Butera IM, Gifford RH, Wallace MT, 2017. Multisensory integration in cochlear implant recipients. Ear Hear. 38, 521–538. 10.1097/AUD.0000000000000435. 28399064 PMC5570631

[R36] Stropahl M, Debener S, 2017. Auditory cross-modal reorganization in cochlear implant users indicates audio-visual integration. NeuroImage Clin. 16, 514–523. 10.1016/j.nicl.2017.09.001. 28971005 PMC5609862

[R37] Sumby WH, Pollack I, 1954. Visual Contribution to Speech Intelligibility in Noise. J. Acoust. Soc. Am 26, 212–215. 10.1121/1.1907309.

[R38] Tremblay C, Champoux F, Lepore F, Théoret H, 2010. Audiovisual fusion and cochlear implant proficiency. Restor. Neurol. Neurosci 28, 283–291. 10.3233/RNN-2010-0498. 20404415

[R39] Zeitler DM, Prentiss SM, Sydlowski SA, Dunn CC, 2024. American cochlear implant alliance task force: recommendations for determining cochlear implant candidacy in adults. Laryngoscope 134, S1–S14. 10.1002/lary.30879. PMC10914083 37435829

